# Changes in Factors Regulating Serum Sodium Homeostasis During Two Ultra-Endurance Mountain Races of Different Distances: 69km vs. 121km

**DOI:** 10.3389/fphys.2021.764694

**Published:** 2021-11-18

**Authors:** Kai Schenk, Simon Rauch, Emily Procter, Katharina Grasegger, Simona Mrakic-Sposta, Hannes Gatterer

**Affiliations:** ^1^Institute of Mountain Emergency Medicine, Eurac Research, Bolzano, Italy; ^2^Department of Sport Science, University of Innsbruck, Innsbruck, Austria; ^3^Department of Anaesthesiology and Intensive Care Medicine, “F. Tappeiner” Hospital, Merano, Italy; ^4^Department of Anaesthesiology and Intensive Care Medicine, BG Klinik Murnau, Murnau, Germany; ^5^National Research Council—Institute of Clinical Physiology (CNR-IFC), Milan, Italy

**Keywords:** mountain running, ultra-marathon, dysnatremia, dehydration, exercise-associated hyponatremia, hyperhydration

## Abstract

Overdrinking and non-osmotic arginine vasopressin release are the main risk factors for exercise-associated hyponatremia (EAH) in ultra-marathon events. However, particularly during ultra-marathon running in mountainous regions, eccentric exercise and hypoxia, which have been shown to modulate inflammation, hormones regulating fluid homeostasis (hypoxia), and oxidative stress, could contribute to serum sodium changes in a dose-dependent manner. To the best of our knowledge, the contribution of these factors, the extent of which depends on the duration and geographical location of the race, has not been well studied. Twelve male participants (11 finishers) of the short (69km, 4,260m elevation-gain) and 15 male participants (seven finishers) of the long (121km, 7,554m elevation-gain) single-stage Südtirol Ultra Sky-Race took part in this observational field study. Venous blood was drawn immediately before and after the race. Analyses included serum sodium concentration, copeptin (a stable marker for vasopressin), markers of inflammation, muscle damage and oxidative stress. Heart rate was measured during the race and race time was obtained from the race office. During the short and the long competition two and one finishers, respectively showed serum sodium concentrations >145mmol/L. During the long competition, one athlete showed serum sodium concentrations <135mmol/L. Only during the short competition percent changes in serum sodium concentrations of the finishers were related to percent changes in body mass (*r*=−0.812, *p*=0.002), total time (*r*=−0.608, *p*=0.047) and training impulse (TRIMP) (*r*=−0.653, *p*=0.030). Data show a curvilinear (quadratic) relationship between percent changes in serum sodium concentration and body mass with race time when including all runners (short, long, finishers and non-finishers). The observed prevalence of hypo- and hypernatremia is comparable to literature reports, as is the relationship between serum sodium changes and race time, race intensity and body mass changes of the finishers of the short race. The curvilinear relationship indicates that there might be a turning point of changes in serum sodium and body mass changes after a race time of approximately 20h. Since the turning point is represented mainly by non-finishers, regardless of race duration slight decrease in body mass and a slight increase in serum sodium concentration should be targeted to complete the race. Drinking to the dictate of thirst seems an adequate approach to achieve this goal.

## Introduction

Ultra-marathon running refers to any running event over marathon distances and can be performed in either a single stage or multi-stage setting (Scheer et al., 2020). In recent years, such competition have become increasingly popular in mountain areas (Stuempfle et al., 2011; Gatterer et al., 2013b, 2020; Mrakic-Sposta et al., 2015; Zanchi et al., 2016; Belinchon-deMiguel et al., 2019, 2021; Hoppel et al., 2019; Nguyen et al., 2021). The most striking features of ultra-mountain marathon running, apart from the long distance, is the difference in altitude that the participants have to overcome, as well as the possible reduced oxygen availability depending on the altitude level. The human body is challenged to the utmost during such races and health problems may occur. Common problems beside others are electrolyte disturbances, hydration imbalances, cardiocirculatory and muscular issues (Gatterer et al., 2013b; Krabak et al., 2017; Knechtle and Nikolaidis, 2018). In the literature, special attention is given to dysnatremia since both dehydration and exercise-associated hyponatremia (EAH) (serum sodium concentration<135mmol/L; Hew-Butler et al., 2017) can lead to serious health issues and even death (Knechtle et al., 2019). The literature on the prevalence of dysnatremia in ultra-endurance events is ample as recently reviewed (Knechtle et al., 2019). Conversely, less data is available on ultra-marathon races performed in the mountains, even though research is progressing fast. During mountain ultra-marathon races, asymptomatic hyponatremia was found in 4–8% of the ultraendurance mountain runners (Page et al., 2007; Arnaoutis et al., 2020), whereas 3% of the participants were hypernatremic (>145mmol/L; Page et al., 2007). Page et al. (2007) furthermore showed that hypernatremia was associated with weight loss whereas hyponatremia with weight gain.

The pathogenesis and clinical representation of EAH have been recently reviewed by Knechtle et al. (2019) and Hew-Butler et al. (2017). Overdrinking and fluid retention due to non-osmotic secretion of arginine vasopressin (AVP) have been identified as the main risk factors (Speedy et al., 2000; Hew-Butler et al., 2017; Knechtle et al., 2019). Overdrinking may stem from the fact that in 1996, the American College of Sports Medicine position stand recommended to consume the maximal amount of fluids that can be tolerated (Convertino et al., 1996). Only in 2007, new recommendation emphasized the risks and consequences of both insufficient and excessive fluid consumption (Sawka et al., 2007). Since then drinking to thirst was considered best practice (Noakes, 2010; Hew-Butler et al., 2017). In addition, factors such as inflammation (linked to AVP regulation; Hew-Butler et al., 2017; Knechtle et al., 2019) and oxidative stress have been linked to the development of dysnatremia (Siegel, 2006; Hew-Butler et al., 2017). Further risk factors include exercise duration >4h, event inexperience, pre-exercise overhydration, low body weight, weight gain during exercise, female sex, and nonsteroidal anti-inflammatory drug use (Almond et al., 2005; Knechtle et al., 2019). Moreover, intrinsic factors like physical strain, perceived exertion, sweat rate and the sensation of thirst and external factors such as race distance, temperature, altitude, humidity, availability and quality of drinks have been identified as contributing factors (O’Connor, 2006; Rosner and Kirven, 2007; Hew-Butler et al., 2008; Schenk et al., 2010; Chlibkova et al., 2018; Knechtle et al., 2019). In this regard, two special features of ultra-mountain marathon running need to be addressed. These events are held at altitude of different levels. Altitude exposure, especially when combined with exercise, induces hypoxemia with profound physiological effects. For instance hypoxia induces oxidative stress (Gatterer et al., 2013a) and inflammation (Pham et al., 2021) and affects hormones regulating fluid homeostasis (Schlittler et al., 2020; Gatterer et al., 2021). A further characteristic is the large proportion of eccentric exercise (downhill running). Similar to hypoxia eccentric exercise induces oxidative stress and inflammation (Bruunsgaard et al., 1997; Lee et al., 2002; Goldfarb et al., 2005). As outlined before inflammation, oxidative stress and dysregulation of hormones involved in fluid homeostasis have been postulated to be involved in the development of dysnatremia. Therefore, these factors must be given special attention, especially in ultra-mountain marathon competitions of varying duration held at altitude (which means different hypoxia dose and eccentric exercise load).

The present work thus aims to describe changes in hormones (e.g., copeptin as a stable marker of AVP), cytokines (e.g., IL-6), oxidative stress and metabolic byproducts (e.g., lactate, ketones) postulated to be linked to sodium regulation during a short (i.e., 69km, 4,260m elevation-gain) and a long (i.e., 121km, 7,554m elevation-gain) mountain ultra-marathon running race with different high altitude exposure levels and eccentric exercise volumes.

## Materials and Methods

### Participants

Participants were recruited by public announcement and by information’s provided by the race office 2months prior to the race. To be eligible, participants had to provide a sports medical health check (including exercise stress testing with ECG monitoring) as required by Italian regulations. Twelve healthy men participating in the short and 15 healthy males participating in the long mountain ultra-run (for details of the competition see below) agreed to take part in this observational field study and provided written informed consent. The informed consent included information’s about EAH and dehydration, yet no specific recommendations were given to the participants prior to the race. Eleven participants completed the short race and seven completed the long race (runners characteristics are shown in Table 1). The study was approved by the Ethical Commission of the Bolzano Hospital, Italy, (No. 57-2015) and was carried out in conformity with the ethical standards of the Declaration of Helsinki.

**Table 1:** Baseline characteristics of the participants and TRIMP during the competitions shown as mean±SD.

	Short distance	Long distance (finishers)	Long distance (non-finishers)	value of p (ES) (short vs. long finishers)	value of p (ES) (long finishers vs. non-finishers)
Age (yr)	42.4 ± 8.8	41.0 ± 10.2	40.8 ± 5.2	0.767 (0.15)	0.952 (0.02)
Body height (m)	1.76 ± 0.64	1.82 ± 0.73	1.76 ± 0.6	0.069 (0.93)	0.118 (0.09)
Body mass (kg)	73.0 ± 7.0	79.0 ± 8.8	69.3 ± 5.4	0.126 (0.75)	0.022 (1.32)
Body mass index (kg/m^2^)	23.6 ± 1.9	23.8 ± 1.3	22.3 ± 1.2	0.860 (0.12)	0.043 (1.20)
VO_2max_ (ml/min/kg)	57.0 ± 6.4	59.3 ± 4.7	59.5 ± 6.3	0.429 (0.41)	0.935 (0.04)
Finishing times (h:min:s)	11:25:53 ± 2:03:31	27:29:15 ± 3:22:41	19:34:47 ± 4:16:33	<0.001 (5.7)	0.002 (2.4)
TRIMP	1,400 ± 145	2,271 ± 321	/	<0.001 (3.50)	/

*Effect size (Cohens’d), ES; maximal oxygen uptake, VO_2max_. n=11 and n=7 for the short and the long distance (finishers), respectively; *n*=4 for TRIMP of the long distance; n=8 for the non-finishers of the long distance*.

### The Race

**Figure 1:**
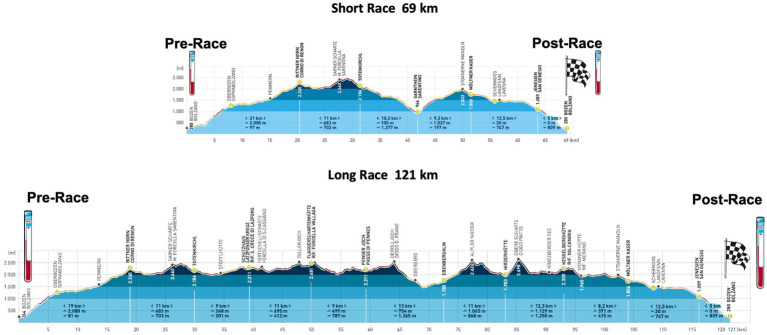
Race profile of the Südtirol Ultra Sky-Race: in the upper part of the figure “the short race” and below “the long race” competition is shown. At pre and post, blood sampling was performed (https://www.suedtirol-ultraskyrace.it/fileadmin/user_upload/pdf/Hoeenprofil_SUSR.pdf).

The single-stage Südtirol Ultra Sky-Race (2015) consisted of a short (69km, 4,260m elevation-gain) and a long (121km, 7,554m elevation-gain) competition (Figure 1). Starting and finishing line was at Bolzano level (262 masl). The long race started in the evening (10pm, overnight race) and the short race in the morning (7am) the next day. On the race days, the mean temperature at Bolzano level (262m) was 26°C (min 22°C, max 30.5°C) during day 1 and 24°C (min 19°C, max 28.5°C) during day 2 with precipitation during the last hours of the long competition.

### Measurements and Instrumentation

Venous blood was collected within 2h before the start of the race and immediately after the race from finishers and within 1h from those who abandoned the race (research staff was present along the course mainly on huts and checkpoints). Food intake before blood collection was not prescribed, as this would have interfered with the participants’ preparation for the competition.

Approximately 14ml of blood was drawn from an antecubital vein and collected in vacutainer tubes (VACUETTE: two Serum, one EDTA). Blood was separated by centrifugation at 3500g for 10min at 4°C, and immediately stored in multiple aliquots at −80°C until being assayed.

Except for the reactive oxygen species (ROS) and copeptin levels, all blood parameters (i.e., white blood cell count, red blood cell count, hemoglobin concentration (Hb), hematocrit (Hct), serum sodium concentration, potassium, bicarbonate, UREA, glucose, ketones, lactate (La), creatine kinase (CK), interleukin-6, osmolality and osmole gap) were analyzed using standard laboratory procedures in the laboratory of the Bolzano hospital. ROS production rate was determined by X-band Electron Paramagnetic Resonance spectroscopy (EPR; E-Scan—Bruker BioSpin, GmbH, Germany), as described in detail elsewhere (Mrakic-Sposta et al., 2014; Vezzoli et al., 2016).

Copeptin levels were determined in duplicate using ELISA kits (MyBioSource, San Diego, United States) according to the manufacturer’s instructions. Inter-assay coefficient of variation was in the range indicated by the manufacturer (CV <10%).

Serum sodium concentration of >145mmol/L were considered to indicate hypernatremia, values between 135 and 145mmol/l as normonatremia and concentrations <135mmol/L as hyponatremia (Noakes et al., 2005).

Bioimpedance analyses (BIA 101 BIVA, AKERN SRL, Montacchielo, Italy) were performed before the race and after the race. All measurements have been performed according to the manufacturer’s guidelines. The subjects were in a supine position with their legs and arms by their sides and values have been registered after a minimum of 5min of rest. Subjects were required to empty their bladder immediately before assuming the supine position. Prior to the measurement, the skin was cleaned with an alcohol solution and four contact electrodes were placed on the dorsal surface of the right hand and foot. For analyses, solely raw values were used (i.e., resistance, reactance and phase angle). Resistance (R) is determined by the body’s resistive (i.e., opposition to flow of current) elements and is related to water content. Reactance (Xc) is determined by the body’s capacitive elements and is associated with cell size and integrity of the cell membranes (Gatterer et al., 2014; Lukaski et al., 2019; Francisco et al., 2020). The phase angle is calculated as arc tangent of Xc/R expressed in degrees and is related to the intracellular water pool and the ratio of extracellular to intracellular volumes (Gatterer et al., 2013c; Gatterer et al., 2014; Francisco et al., 2020).

Throughout the race, participants were equipped with heart rate (HR, Polar and Garmin) monitors. Data were lost for three participants during the long run because of inadequate battery life. Official race times of the participants were obtained from the race office.

### Pre-race Exercise Testing and Race Effort

Exercise testing was performed on a treadmill up to 2weeks before the race as described in detail elsewhere (Gatterer et al., 2020). Briefly, the running test protocol included the following speed and incline increases: 2min at 5km/h walking speed with an incline of 5%, 2min at 5km/h walking speed with an incline of 10%, 1min at 6km/h walking speed with an incline of 10%, then the incline was increased by 2% per minute up to 20%, then participants started running and running speed was increased by 1km/h per min up to exhaustion. Training impulse (TRIMP) was calculated as: LOW + MOD x 1.5+HI x 2+VHI x 3, where LOW corresponds to the time spent in the low intensity zone (heart rate below the ventilator threshold (VT) 1), MOD to the time spent in the moderate intensity zone (heart rate between VT1 and [(HR-VT2−HR-VT1)/2+HR-VT1]), HI to the time spent in the high intensity zone (heart rate values between [(HR-VT2−HR-VT1)/2+HR-VT1] and VT2) and VHI to the time spent in the very high intensity zone (heart rate above VT2) (Gatterer et al., 2020).

### Statistical Analysis

The sample size depended on participants’ willingness to respond to our call and whether participants were able to complete the race. Data were analyzed using IBM SPSS Statistics 26. Paired t-tests were used to analyze changes in the course of the short and long competition as well as of the combined race distances. For the comparison between the short and the long run, unpaired t-tests were used. Pearson correlation analyses were used to test for significant relationships between percent changes in serum sodium concentration and variables putatively related to sodium changes [correlation coefficient of *r*=0.1 indicates a small, *r*=0.3 a medium and *r*=0.5 a large effect size (Cohen, 1988)]. Additionally, curve estimation was used to check for potential quadratic relationships for data of all participants (finishers, non-finishers, short and long competition). Data are presented as mean±SD and information on effect size (ES, Cohen’s d) was included, where *d*=0.2 indicates a small, *d*=0.5 a medium and *d*=0.8 a large effect (Cohen, 1988). Statistical significance was set at *p*<0.05.

## Results

During the short competition two out of the 11 finishers (18.2%) showed serum sodium concentrations >145mmol/L (148 and 147mmol/L). The single non-finisher also demonstrated a serum sodium concentration of >145mmol/L (146mmol/L). No cases of hyponatremia were registered. During the long competition, one athlete out of the seven finishers (14.3%) was hyponatremic (134mmol/L) and one hypernatremic (147mmol/L). All eight non-finishers showed serum sodium concentration in the normal range. Fluid intake per hour was 612.4±187.6ml/h during the short and 422.3±197.8ml/h during the long competition (*p*=0.057). Table 2 displays changes in putative factors regulating serum sodium homeostasis as well as standard clinical parameters during the short and the long competition. Whereas marked changes were registered in most of the parameters from before to after both races, different changes between the short and long competition were only found for reactance and phase angle of the bioimpedance analyses, red blood cell count, Hb and La concentration and CK (Table 2). The four cases of hypernatremia showed a loss in body mass from 76.5±3.9 to 72.4±3.3kg (*p*=0.002) and increases in ROS (0.179±0.004 to 0.200±0.142μmol/min; *p*=0.020), IL-6 (2.25±0.25 to 33.10±10.56pg./ml; *p*=0.010), copeptin (8.4±2.5 to 24.7±9.3pmol/L; *p*=0.040) and WBC count (6.9±3.0 to 15.9±5.4 10^3^/μl; *p*=0.005).

**Table 2:** Changes in measured variables of the finishers during two ultra-endurance mountain races of different distances: 69km, 4,260m elevation-gain vs. 121km, 7,554m elevation-gain (*n*=11 and *n*=7 for the short and the long distance, respectively).

	Short distance			Long distance			*p* (ES)
	Pre	Post	%changes	Pre	Post	%changes	Differences of %changes between short and long
Body mass (kg)	73.0 ± 7.0	70.7 ± 6.7	−3.1 ± 1.3^*^	79.0 ± 8.8	76.9 ± 8.6	−2.7 ± 2.0^*^	0.553 (0.24)
Resistance (Ω)	450.9 ± 42.2	454.3 ± 41.4	0.8 ± 3.5	463.1 ± 30.4	450.2 ± 25.2	−2.5 ± 7.2	0.206 (0.58)
Reactance (Ω)	54.8 ± 6.4	60.6 ± 6.9	10.7 ± 4.3^*^	57.3 ± 4.0	56.0 ± 4.1	−1.7 ± 11.7	**0.005** (1.41)
Phase angle (°)	6.95 ± 0.66	7.62 ± 0.70	9.8 ± 2.7^*^	7.04 ± 0.26	7.20 ± 0.41	2.2 ± 4.8	**0.001** (1.95)
WBC count (10^3^/μl)	6.96 ± 1.87	16.79 ± 4.04	147.5 ± 59.2^*^	6.38 ± 1.18	14.69 ± 2.45	131.6 ± 21.7^*^	0.434 (0.36)
RBC count (10^6^/μl)	5.02 ± 0.33	5.23 ± 0.40	4.1 ± 4.1^*^	4.88 ± 0.33	4.81 ± 0.43	−1.7 ± 4.1	**0.010** (1.41)
Hb (g/dl)	14.8 ± 0.7	15.4 ± 0.9	3.6 ± 3.8^*^	14.5 ± 1.0	14.3 ± 1.0	−1.8 ± 3.3	**0.006** (1.52)
Hct (%)	44.7 ± 2.1	45.7 ± 2.4	2.2 ± 4.8	43.9 ± 2.2	42.7 ± 3.1	−2.7 ± 5.6	*0.067* (0.94)
Sodium (mmol/L)	142.9 ± 1.9	143.7 ± 2.1	0.6 ± 2.1	142.0 ± 1.7	142.6 ± 4.2	0.4 ± 2.7	0.870 (0.08)
Potassium (mmol/L)	3.96 ± 0.21	3.92 ± 0.42	−0.9 ± 9.1	3.97 ± 0.42	3.89 ± 0.23	−1.4 ± 9.7	0.911 (0.05)
Bicarbonate (mmol/L)	26.1 ± 2.2	23.1 ± 2.6	−10.9 ± 13.2^*^	25.1 ± 2.0	21.7 ± 2.8	−13.3 ± 12.4^*^	0.705 (0.19)
UREA (mg/dl)	30.3 ± 5.8	48.4 ± 6.0	65.2 ± 37.5^*^	36.7 ± 12.6	74.3 ± 31.3	106.6 ± 60.4^*^	*0.090* (0.82)
Glucose (mg/dl)	99.6 ± 8.6	117.4 ± 22.6	18.3 ± 23.3^*^	97.9 ± 5.3	109.6 ± 12.0	12.2 ± 12.8^*^	0.541 (0.32)
Ketones (mmol/L)	0.21 ± 0.05	0.40 ± 0.12	110.6 ± 131.3^*^	0.29 ± 0.11	0.57 ± 0.17	132.9 ± 122.0^*^	0.724 (0.18)
Lactate (mmol/L)	1.69 ± 059.	3.72 ± 1.76	142.2 ± 134.9^*^	1.46 ± 0.50	1.75 ± 0.76	29.3 ± 66.8	**0.032** (1.06)
Creatine kinase (U/L)	180.2 ± 102.4	1,741.7 ± 1,269.6	1,012.4 ± 827.4^*^	151.0 ± 57.3	5,083.6 ± 3,782.8	3,304.2 ± 2,286.4^*^	**0.007** (1.33)
Interleukin-6 (pg/ml)	1.92 ± 0.51	51.05 ± 19.55	2,769.1 ± 1,315.9^*^	2.44 ± 0.88	61.93 ± 58.12	2,208.8 ± 1,216.7^*^	0.378 (0.44)
Copeptin (pmol/L)	7.66 ± 2.76	27.82 ± 12.76	313.7 ± 248.6^*^	8.75 ± 2.13	26.28 ± 13.71	214.6 ± 155.5^*^	0.362 (0.48)
Reactive Oxygen Species (μmol/min)	0.172 ± 0.010	0.205 ± 0.013	19.8 ± 6.2^*^	0.174 ± 0.014	0.206 ± 0.008	19.2 ± 9.1^*^	0.870 (0.08)
Osmolality (mOsm/kg)	286.1 ± 3.0	292.2 ± 5.8	2.1 ± 2.4^*^	284.4 ± 3.7	293.6 ± 11.8	3.2 ± 3.7	0.465 (0.35)
Osmole gap (mOsm/kg)	9.6 ± 5.2	10.9 ± 4.1	28.4 ± 54.2	8.8 ± 1.7	9.9 ± 3.8	19.3 ± 50.1	0.724 (0.17)

*Effect size (Cohens’d), ES; hematocrit, Hct; hemoglobin concentration, Hb; red blood cell count, RBC; with blood cell count, WBC. Bold indicates a p<0.05; Italic indicates 0.05<p<0.10*.

* *Indicates significant differences from pre to post*.

During the short competition percent changes in serum sodium concentrations were negatively related to percent changes in body mass (*r*=−0.812, *p*=0.002), total time (*r*=−0.608, *p*=0.047) and TRIMP (*r*=−0.653, *p*=0.030) and positively related to UREA (*r*=0.612, *p*=0.046). Correlations between percent changes in serum sodium concentrations and percent changes in bicarbonate (*r*=−0.572, *p*=0.066) and osmolality (*r*=0.582, *p*=0.060) did not reach statistical significance even though effect size was large. During the long race, no significant correlation between percent changes in serum sodium concentration and any other measured variable was detected. When combining data of the long and the short run, significant correlations between percent changes in serum sodium concentration and changes in body mass (*r*=−0.702, *p*=0.001) and osmolality (*r*=0.600, *p*=0.008) were recorded. Table 3 displays differences in changes between finishers and non-finishers of the long competition. Data show that body mass, UREA, CK and osmolality differed between finishers and non-finishers. When including all runners (short, long, finishers and non-finishers) percent changes in serum sodium concentration and body mass showed a more curvilinear (quadratic) rather than linear relationship with race time (Figure 2).

**Table 3:** Changes in measured variables of the finishers and non-finishers of the long mountain ultra-marathon running race (*n*=7 and *n*=8 for the finishers and the non-finishers, respectively).

	Finishers			Non-finishers			*p* (ES)
	Pre	post	%changes	Pre	Post	%changes	Differences of %changes between finishers and non-finishers
Body mass (kg)	79.0 ± 8.8	76.9 ± 8.6	−2.7 ± 2.0^*^	69.3 ± 5.4^#^	68.9 ± 4.8	−0.6 ± 1.5	**0.041** (1.19)
Resistance (Ω)	463.1 ± 30.4	450.2 ± 25.2	−2.5 ± 7.2	473.9 ± 23.3	458.8 ± 33.2	−3.2 ± 4.8	0.827 (0.11)
Reactance (Ω)	57.3 ± 4.0	56.0 ± 4.1	−1.7 ± 11.7	58.1 ± 5.8	58.0 ± 6.4	−0.1 ± 7.5	0.752 (0.16)
Phase angle (°)	7.04 ± 0.26	7.20 ± 0.41	2.2 ± 4.8	6.98 ± 0.52	7.16 ± 0.40	2.9 ± 5.8	0.809 (0.13)
WBC count (10^3^/μl)	6.38 ± 1.18	14.69 ± 2.45	131.6 ± 21.7^*^	5.90 ± 1.25	11.65 ± 2.14	104.1 ± 55.1^*^	0.238 (0.66)
RBC count (10^6^/μl)	4.88 ± 0.33	4.81 ± 0.43	−1.7 ± 4.1	5.09 ± 0.32	5.08 ± 0.34	−0.1 ± 4.9	0.513 (0.35)
Hb (g/dl)	14.5 ± 1.0	14.3 ± 1.0	−1.8 ± 3.3	14.9 ± 0.9	14.5 ± 0.7	−2.2 ± 5.9	0.881 (0.08)
Hct (%)	43.9 ± 2.2	42.7 ± 3.1	−2.7 ± 5.6	44.6 ± 2.2	44.3 ± 2.4	−0.6 ± 4.9	0.461 (0.40)
Sodium (mmol/L)	142.0 ± 1.7	142.6 ± 4.2	0.4 ± 2.7	142.5 ± 2.9	140.6 ± 2.3	−1.3 ± 1.8	0.170 (0.74)
Potassium (mmol/L)	3.97 ± 0.42	3.89 ± 0.23	−1.4 ± 9.7	3.94 ± 0.24	3.84 ± 0.38	−2.5 ± 7.9	0.817 (0.12)
Bicarbonate (mmol/L)	25.1 ± 2.0	21.7 ± 2.8	−13.3 ± 12.4^*^	27.4 ± 2.4	25.1 ± 2.2	−8.1 ± 5.2^*^	0.336 (0.55)
UREA (mg/dl)	36.7 ± 12.6	74.3 ± 31.3	106.6 ± 60.4^*^	37.1 ± 10.0	45.4 ± 15.9	23.8 ± 45.0	**0.010** (1.55)
Glucose (mg/dl)	97.9 ± 5.3	109.6 ± 12.0	12.2 ± 12.8^*^	95.8 ± 8.6	112.1 ± 23.5	16.8 ± 19.4	0.597 (0.28)
Ketones (mmol/L)	0.29 ± 0.11	0.57 ± 0.17	132.9 ± 122.0^*^	0.19 ± 0.04^#^	0.65 ± 0.70	237.5 ± 343.0	0.459 (0.41)
Lactate (mmol/L)	1.46 ± 0.50	1.75 ± 0.76	29.3 ± 66.8	1.34 ± 0.63	1.46 ± 0.64	20.8 ± 56.1	0.792 (0.14)
Creatine kinase (U/L)	151.0 ± 57.3	5,083.6 ± 3,782.8	3,304.2 ± 2,286.4^*^	201.3 ± 152.3	2,218.9 ± 2,045.3	1,306.8 ± 1,151.0^*^	**0.048** (1.10)
Interleukin-6 (pg/ml)	2.44 ± 0.88	61.93 ± 58.12	2,208.8 ± 1,216.7^*^	2.08 ± 0.66	24.3 ± 11.88^*^	1,157.3 ± 722.6^*^	*0.059* (1.05)
Copeptin (pmol/L)	8.75 ± 2.13	26.28 ± 13.71	214.6 ± 155.5^*^	4.57 ± 2.38^#^	25.25 ± 6.50^*^	599.6 ± 469.9^*^	*0.060* (1.10)
Reactive Oxygen Species (μmol/min)	0.174 ± 0.014	0.206 ± 0.008	19.2 ± 9.1^*^	0.172 ± 0.013	0.199 ± 0.018^*^	15.8 ± 8.5^*^	0.476 (0.39)
Osmolality (mOsm/kg)	284.4 ± 3.7	293.6 ± 11.8	3.2 ± 3.7	287.6 ± 5.0	285.8 ± 1.7	−0.6 ± 1.8	**0.021** (1.31)
Osmole gap (mOsm/kg)	8.8 ± 1.7	9.9 ± 3.8	19.3 ± 50.1	11.1 ± 4.3	10.4 ± 2.6	8.6 ± 50.0	0.687 (0.21)

*Effect size (Cohens’d), ES; hematocrit, Hct; hemoglobin concentration, Hb; red blood cell count, RBC; with blood cell count, WBC. Bold indicates a p<0.05; Italic indicates 0.05<p<0.10*.

* *Indicates significant changes from pre to post*.

# *Indicates significant pre-differences between groups*.

**Figure 2:**
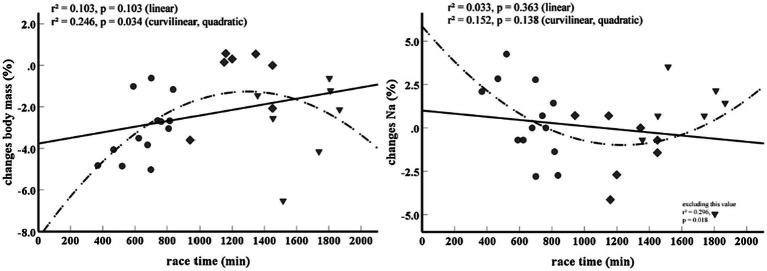
Curvilinear (quadratic) relationship between **(A)** race time and percent changes in body mass and **(B)** race time and percent changes in serum sodium concentration. At the bottom right, Figure 1B shows the outcome of the analysis when excluding one outlier. Circles represent finishers of the short race, rhombus non-finishers of the long race and triangles finishers of the long race. Na, serum sodium concentration.

## Discussion

The main findings of this investigation were that during the short ultra-endurance mountain race no case of hyponatremia was observed whereas during the long race one out of seven was hyponatremic, yet without showing any symptoms potentially related to serum sodium aberrations (e.g., malaise, headache; Knechtle et al., 2019). Conversely, during both races cases of hypernatremia were recorded. Serum sodium concentration changes were related to race time, race intensity (i.e., TRIMP) and body mass changes during the short race whereas none of the assessed parameters where related to the long race indicating a threshold where other factors may determine serum sodium aberrations.

It is well established that drinking habits in conjunction with hormonal dysregulation, race duration and race intensity are the main factors linked to serum sodium alterations (Rosner, 2019). Present data support these findings during the short race showing a relationship between serum sodium changes and race time, race intensity and body mass changes. Interestingly no such relationship was detected for the long race. It is important to mention that hyponatremia is a condition linked to death during endurance races (Hew-Butler et al., 2017; Rosner, 2019). There was only one single case of asymptomatic hyponatremia occurring in an athlete competing in the long race. When having a closer look at this athlete, data indicate similar mechanisms as reported in the literature (Hew-Butler et al., 2017; Rosner, 2019). The athlete ran for 30h and showed only small changes in body mass (~−0.5kg), whereas Hb concentration and Hct indicated hemodilution (14.9g/dl and 46.7% before the race and 13.8g/dl and 41.8% after the race, respectively). This athlete also showed the highest values for ROS production corrected for plasma volume changes (0.258 vs. 0.203±0.017) and one of the highest post exercise copeptin values (37.2 vs. 26.7±12.3). High levels of copeptin and AVP during and at the end of ultra-marathon race (160km) despite decreases in [Na^+^] (confirming non-osmotic simulation of AVP secretion) are in agreement with Hew Butler et al. (2011). Obviously, this is a single case observation prone to errors, nonetheless it supports both well-established research findings (Page et al., 2007; Rosner, 2019), as well as recent postulations stating that oxidative stress is involved in hyponatremia development (Hew-Butler et al., 2017). Changes in values of the four hypernatremia cases were similar to the ones recorded for the normonatremic competitors (finishers) of both races. This was to be expected because the serum sodium values of the hypernatremic cases were only slightly above the threshold for hypernatremia of >145mmol/L.

Comparison of the outcomes of the short and the long run (Table 2) shows that race distance did not affect ROS production, interleukin-6, copeptin and serum sodium concentrations. This indicates that different hypoxic doses and eccentric exercise volumes did not have a large effect on those changes, at least in these competitions. Since muscle damaging downhill running is associated with higher CK values (Gatterer et al., 2013b), the higher values found after the long run confirm a higher proportion of eccentric exercise compared to the short race. Concerning hypoxia dose, no information is available, as oxygen saturation was not measured. However, at altitude levels of around 2,000–2,500m (where much of the racing during the long run took place, Figure 1), oxygen saturation of around 90% may be expected during low intensity exercise, with individual values even around 80% (Wiseman et al., 2013). Nonetheless, it must be recognize that the physiological stress induced by this relatively low hypoxic dose may have been limited. The comparison between race distances furthermore shows that reactance and phase angle of the bioimpedance analysis are differently affected. According to recent literature, increases in phase angle indicate fluid shifts to the intracellular compartment (Francisco et al., 2020). Thus, data seem to indicate that during the short run fluid shifts to the intracellular compartment are more pronounced compared to the long run. Differences in exercise intensity between the races (Gatterer et al., 2020) may to some extent explain these findings, since race intensity was reported to be related to leg edema formation (Wilcock et al., 2006; Bracher et al., 2012). A further finding was that lactate concentration was lower after the long run. Single lactate measurements must be interpreted with caution, as they may only represent the activity prior to the measurement. However, they might also reflect a slower running speed (Gatterer et al., 2020) or muscle/liver glycogen depletion (Segal and Brooks, 1979) during the long run. No differences were observed for ketones, indicating similar free-fatty acid breakdown (Evans et al., 2017).

An interesting observation was that the percentage changes in serum sodium concentration and body mass showed a curvilinear (quadratic) rather than a linear relationship with race time when data from all athletes (short and long distance, finishers, and non-finishers) were included in the analysis (Figure 2). Such a curvilinear relationship was recently reported for cardiac biomarkers and race duration in marathon running (Traiperm et al., 2021). Additionally, cardiac biomarkers seemed less elevated the longer the running time in a 118km ultra-mountain race (Martinez-Navarro et al., 2019). It was speculated that short running times may prevent excessive and prolonged volume overload and myocyte stretch (Shave et al., 2007; Serrano-Ostariz et al., 2011) whereas slow running times reflect low running pace and thus less overall strain (Traiperm et al., 2021). In the present investigation, the fastest runners of the short run showed the highest serum sodium increases and body mass reductions. With increasing race time, serum sodium concentrations seem to decrease whereas less of a body mass loss was apparent. This is in accordance to reports showing that race duration and body mass gain (or less decrease) are risk factors for hyponatremia (Rosner, 2019). Furthermore it supports the notion that the fastest runners lose the most body mass (Noakes, 2010). Interestingly, during the long run the opposite pattern was found. There the fastest runners showed the highest decreases in serum sodium concentrations and the lowest decreases in body mass. The longer the performance time the more the serum sodium increased and the more the body mass decreased. The area around the nadir (Figure 2) is identified at about 20h of performance time and is mostly represented by runners that did not finish the race. In addition to showing that the shortest and the longest race times might be associated with both increases in serum sodium concentrations and losses of body mass, this graph indicates that too much of a serum sodium concentration decrease and too less of a body mass reduction are associated with a higher drop-out risk. Certainly, this observation has to be confirmed in further studies and it must be emphasized that effect sizes of the correlations were rather low, and no causal effect can be inferred. Furthermore, the reasons for a race being abandoned can be numerous (e.g., pain, fatigue, and gastrointestinal problems) as can the factors that determine race time.

### Methodological Considerations

Some limitations of this observational field study have to be acknowledged. A major limitation is the limited sample size and the fact that overall changes in serum sodium concentration have been low, which limits the conclusiveness of the present data. The sample size depended on participants’ willingness to respond to our call and thus was not modifiable. The low sample size, mainly of the long competition, may to some extent explain why no significant correlation during the long race was recorded between percent changes in serum sodium concentration and any other measured variable. Even though statistically not significant, large effects were found for percent changes in serum sodium concentration and body mass (*r*=−0.652, *r*=0.113), ketones (*r*=0.706, *p*=0.077), osmolality (*r*=0.644, *p*=0.118), and copeptin (*r*=−0.628, *r*=0.131). Since nonparametric testing (Mann–Whitney test, Wilcoxon, and Spearman analyses) may be more robust when analyzing small sample sizes, we performed such analyses and found similar results, except for slight different p- and r-values. However, it should be mention that in the comparison between finishers and non-finishers of the long race, IL-6 and copeptin became significant (*p*<0.05). Moreover, the correlation coefficient for changes in serum sodium concentration and total race time of the short race was slightly lower when using Spearman analysis, *r*=−0.566, *p*=0.069. A further limitation is that data on the volume of fluid intake were obtained from personal assessments at the end of the race, which is prone to errors. In addition, diet before and during the race as well as the type of fluid intake were not assessed. However, as the participants adopted their normal race routines, this procedure should reflect real-life scenarios. It must also be considered that EAH may develop independently of body mass changes (Knechtle et al., 2019), yet body mass changes, particularly body mass gain, are still considered a surrogate for serum sodium changes (Knechtle et al., 2019). Moreover, some HR data and thus race intensity were not available because of inadequate battery life of the heart rate sensors.

In conclusion, one out of seven finishers (14%) showed asymptomatic hyponatremia after the long race whereas hypernatremia was present after the long (14%) and the short race (18%). Race duration and intensity as well as body mass changes were related to serum sodium concentrations changes during the short race. No such correlation was recorded for the long race, yet the single hyponatremic case showed hemodilution, minor body mass loss and high levels of ROS and copeptin. From a practical point of view, the observed curvilinear relationships suggest that regardless of race duration (which determines hypoxia dose and eccentric exercise load) a slight decrease in body mass and a slight increase in serum sodium concentration should be targeted to complete the race. On the other hand, to compete fast in the long run, permitting only small changes seem beneficial. Excessive increases in body mass and reductions in serum sodium concentrations should be avoided in any circumstance. According to the literature, drinking to the dictate of thirst seems an adequate approach to achieve this goal also during ultra-marathon competitions in mountain areas.

## Data Availability Statement

The raw data supporting the conclusions of this article will be made available by the authors, without undue reservation.

## Ethics Statement

The studies involving human participants were reviewed and approved by the Ethical Commission of the Bolzano Hospital, Italy. The patients/participants provided their written informed consent to participate in this study.

## Author Contributions

KS designed, planned, and implemented the study. KS, SR, EP, KG, SM-S, and HG performed the measurements. HG and KS drafted the manuscript and analyzed the data. SR, EP, KG, and SM-S contributed in writing the manuscript. All authors contributed to the article and approved the submitted version.

## Conflict of Interest

The authors declare that the research was conducted in the absence of any commercial or financial relationships that could be construed as a potential conflict of interest.

## Publisher’s Note

All claims expressed in this article are solely those of the authors and do not necessarily represent those of their affiliated organizations, or those of the publisher, the editors and the reviewers. Any product that may be evaluated in this article, or claim that may be made by its manufacturer, is not guaranteed or endorsed by the publisher.
